# Diagnostic accuracy of high-temporal resolution whole heart coronary magnetic resonance angiography at 3.0 Tesla

**DOI:** 10.1186/1532-429X-11-S1-P154

**Published:** 2009-01-28

**Authors:** Nagata Motonori, Hajime Sakuma, Nanaka Ishida, Shingo Kato, Hiroshi Nakajima, Masaki Ishida, Kakuya Kitagawa, Katsuya Onishi, Masaaki Ito, Kan Takeda

**Affiliations:** Mie University hospital, Tsu, Mie, Japan

**Keywords:** Significant Coronary Artery Disease, Average Heart Rate, Branch Vessel, Image Quality Score, Acquisition Window

## Introduction

30 T whole heart coronary MR angiography permits acquisition of coronary arterial images with improved signal to noise ratio and high spatial resolution. The use of a patient-specific, narrow acquisition window in the cardiac cycle can reduce motion blurring on coronary MR angiography, and may allow for improved delineation of distal coronary arteries and branch vessels.

## Purpose

The purpose of this study was to determine the image quality and diagnostic accuracy of 3.0 T whole-heart coronary MR angiography acquired with a subject-specific, narrow acquisition window in the cardiac cycle.

## Methods

Free breathing whole heart coronary MR angiograms were acquired in 32 patients with suspected coronary artery disease by using a 3.0 T MR imager and 6 channel cardiac coil system. After administration of gadolinium contrast medium, navigator-echo gated, 3-dementional TFE images were obtained with T2 preparation, fat saturation, TR/TE/FA of 4.2 ms/2.1 ms/20 degree and SENSE factor of 2. A narrow data acquisition window in the cardiac cycle was used by monitoring motion of the coronary arteries on cine MRI in each patient. MRA data were acquired during diastole in 22 patients (averaged heart rate = 65.5 ± 8.2 beat/min, acquisition window = 63 ± 25 ms), and during systole in 10 patients (averaged heart rate = 75.1 ± 10.3 beats/min, acquisition window = 36.2 ± 6.2 ms). Image quality was classified on a 4-point scale (1 = poor, 2 = moderate, 3 = good, 4 = excellent). X-ray coronary angiograms were obtained in 11 patients within 2 weeks from MR study. Significant coronary artery disease was defined as a diameter reduction of ≥ 50 % in coronary arteries with a reference of ≥ 2 mm on X-ray coronary angiography. MRA images were interpreted by 2 independent observers by using a sliding multiplanar reformat reconstruction method, and disagreement between 2 observers was settled by a consensus reading.

## Results

**Figure 1:**
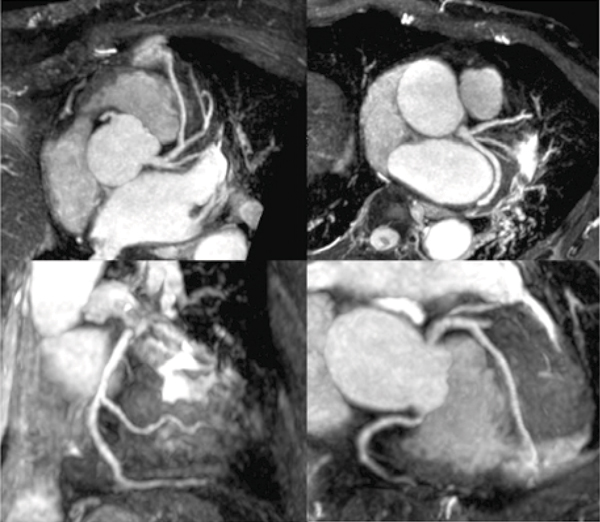
**Sliding partial MIP images of 3 T whole heart coronary MRA acquired with a patient-specific narrow acquisition window (50 ms) in the cardiac cycle**.

**Figure 2:**
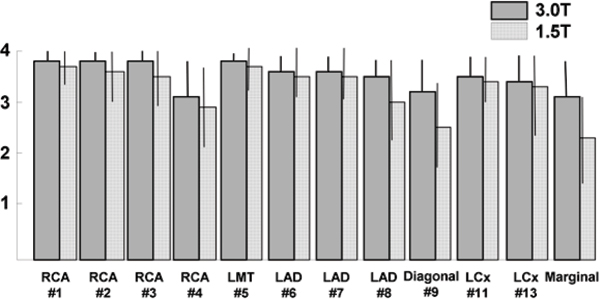
**Averaged image quality score of 3 T whole heart coronary MRA compared with 1.5 MRA**.

Acquisition of whole heart coronary MR angiography was completed in all 32 patients with averaged imaging time of 11.4 ± 4.5 minutes. Excellent average image quality scores (≥ 3.8) were observed in the proximal and mid portion of the arteries (RCA #1–3, LMT#5, LAD #6–7, LCX #11) (Figure 1). In addition, high image quality scores (≥ 3.0) were achieved in the distal segments and branch vessels as well (RCA#4, LAD#8–9, LCX#12–13) (Figure 2). On a vessel-based analysis, the sensitivity, specificity, positive and negative predictive value and accuracy of whole heart coronary MR angiography for the detection of significant coronary artery disease was 83% (95% confidence interval [CI] 36–99%), 96% (95%CI 79–100%), 83% (95%CI 36–99%), 96% (95%CI 79–100%), and 94% (95%CI 86–100%), respectively.

## Conclusion

30 T free-breathing whole heart coronary MR angiography acquired with a subject-specific, narrow acquisition window in the cardiac cycle permits improved visualization of coronary arteries including distal segments and branch vessels. High negative predictive value found in this study indicates the value of 3.0 T coronary MRA for screening significant coronary artery disease.

